# Common mechanisms underlying axonal transport deficits in neurodegenerative diseases: a mini review

**DOI:** 10.3389/fnmol.2023.1172197

**Published:** 2023-04-24

**Authors:** Xiaoman Yang, Zhuoran Ma, Piaopiao Lian, Yan Xu, Xuebing Cao

**Affiliations:** Department of Neurology, Union Hospital, Tongji Medical College, Huazhong University of Science and Technology, Wuhan, China

**Keywords:** axonal transport deficit, Alzheimer’s disease, amyotrophic lateral sclerosis, Parkinson’s disease, Huntington’s disease

## Abstract

Many neurodegenerative diseases including Alzheimer’s disease, Parkinson’s disease, and amyotrophic lateral sclerosis are characterized by the accumulation of pathogenic proteins and abnormal localization of organelles. These pathological features may be related to axonal transport deficits in neurons, which lead to failures in pathological protein targeting to specific sites for degradation and organelle transportation to designated areas needed for normal physiological functioning. Axonal transport deficits are most likely early pathological events in such diseases and gradually lead to the loss of axonal integrity and other degenerative changes. In this review, we investigated reports of mechanisms underlying the development of axonal transport deficits in a variety of common neurodegenerative diseases, such as Alzheimer’s disease, amyotrophic lateral sclerosis, Parkinson’s disease and Huntington’s disease to provide new ideas for therapeutic targets that may be used early in the disease process. The mechanisms can be summarized as follows: (1) motor protein changes including expression levels and post-translational modification alteration; (2) changes in microtubules including reducing stability and disrupting tracks; (3) changes in cargoes including diminished binding to motor proteins. Future studies should determine which axonal transport defects are disease-specific and whether they are suitable therapeutic targets in neurodegenerative diseases.

## 1. Introduction

**Figure 1:**
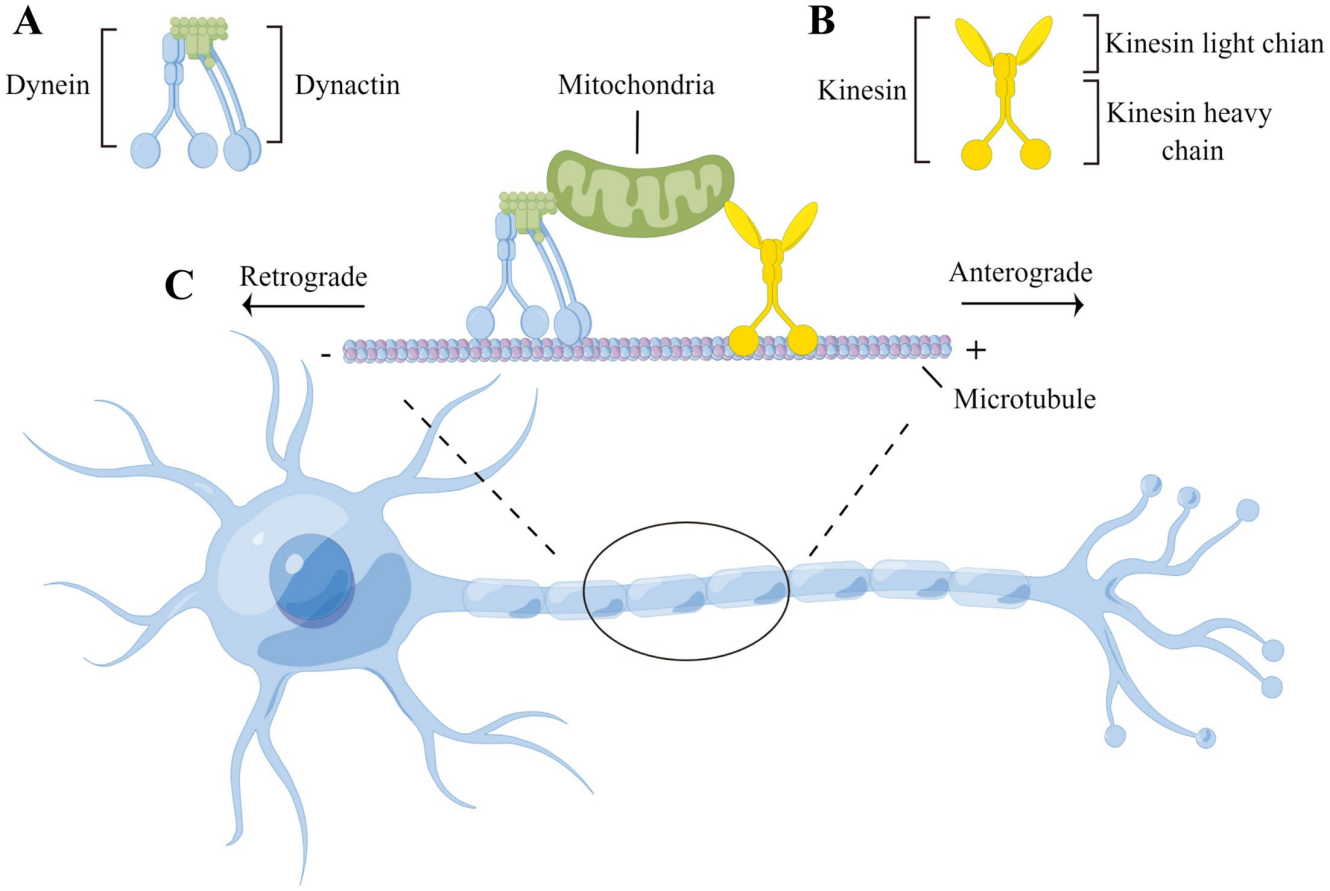
Microtubule-based neuronal axonal transport. **(A)** Dynein transport complex consists of dynein’s heavy, medium, medium-light, and light chains and dynactin, which move along the axon to transport cargoes in a retrograde direction (i.e., toward the minus end of the microtubule and cell body); **(B)** Kinesin consists of two heavy chains and two light chains, which transport cargoes in a anterograde direction along the axon (i.e., toward the plus end of the microtubule and synaptic terminals); **(C)** The heavy chains of both kinesin and dynein have a motor structural domain that binds to the microtubule, hydrolyzes ATP, and propels cargo along the microtubule track. The cargoes are attached to motor proteins via kinesin’s light chain or dynactin.

Neurons are polarized and morphologically complex cells that possess cytoplasmic extensions known as dendrites and axons. Most proteins and organelles are firstly synthesized in the cell body and subsequently transported along axons to specific cellular regions such as synaptic terminals to perform their functions. Axonal transport maintains long-distance communication between the cell body and synaptic terminal, allowing neurons to provide essential components for distal axons and terminals and recycle proteins to the cell body. This long-distance transport mechanism consists of the following two main components: microtubules, which serve as transport tracks, and motor proteins, which are responsible for movement along microtubules. On microtubules, kinesins promote movement to microtubule plus ends, mediating the anterograde flow of organelles and mRNA to the synaptic terminals of neurons and meeting local energy demands. In contrast, cytoplasmic dynein travels towards microtubule minus ends, mediating the retrograde transport of cargoes from synaptic terminals towards the cell body, clearing misfolded proteins, and promoting the intracellular transport of distal neurotrophic signals to the cell body (Figure 1).

Abnormal aggregation of cellular components is a major pathological feature of many neurodegenerative diseases. Abnormal aggregation of tau is a central pathogenic mechanism in Alzheimer’s disease (AD) and related disorders (Zhang et al., 2021). Pathological α-synuclein (α-Syn) is a major component of Lewy bodies (LB) in Parkinson’s disease (PD) (Wong and Krainc, 2017). Furthermore, in patients with amyotrophic lateral sclerosis (ALS), neurofilaments (NFs) and TAR DNA binding protein 43 (TDP-43) tend to accumulate in the cytoplasm (Yu et al., 2020; Kumar et al., 2021). Neuronal transport dysfunction, especially axonal transport impairment, may affect the intracellular localization and degradation of aforementioned proteins, leading to pathogenic increases in aggregation. Additionally, pathological proteins may impair axonal transport functions of neurons as well (Folwell et al., 2010; Wood et al., 2021), driving the pathological progression of neurodegenerative diseases. Considering impaired axonal transport may be an early pathological event in many neurodegenerative diseases (Millecamps and Julien, 2013), this review summarizes the main mechanisms by which axonal transport malfunctions in common neurodegenerative diseases and explores the relationship between pathological proteins and axonal transport defects, potentially facilitating the identification of therapeutic targets that may be used early in the disease process.

## 2. Axonal transport defects in neurodegenerative diseases

### 2.1. Alzheimer’s disease

AD is one of the most common neurodegenerative diseases, which mainly manifests as progressive cognitive dysfunction. Abnormal aggregation of tau within neurons and formation of amyloid β-protein (Aβ) are two core pathological hallmarks of the disease (Zhang et al., 2021).

Numerous studies have shown abnormal mitochondrial distribution in the neurons of human AD cases (Wang et al., 2009; Pickett et al., 2018). Under normal conditions, new mitochondria fill axons and synaptic terminals via kinesin-based anterograde transport, and damaged mitochondria are retrogradely transported to the cell body for degradation by cytoplasmic dynein. This cycle helps maintain mitochondrial homeostasis within neurons (Sheng and Cai, 2012; Lin et al., 2017). It has been shown in the transgenic rTg4510 mouse model that mitochondria are not evenly distributed throughout neurons, particularly in neurites. Further, large axonal and dendritic segments are completely devoid of mitochondria in neurons of human AD brains (Kopeikina et al., 2011), implying the relevance of axonal transport defect to the disrupted mitochondrial distribution.

Primary neurons isolated from transgenic Tg2576 mice expressing the amyloid precursor protein (APP) exhibit decreased anterograde mitochondrial movement, a pathological process that has been suggested by some investigators to be mediated by the accumulation of oligomeric Aβ (Calkins et al., 2011). Similarly, in a transgenic *Drosophila* model, Aβ42 has led to intracellular mitochondrial mis-localization, reduction of the number of mitochondria within axons and dendrites, disrupted anterograde axonal transport and intracellular mitochondrial accumulation (Iijima-Ando et al., 2009). The precise mechanism by which Aβ affects mitochondrial axonal transport is unclear. Using Aβ-treated primary neurons and the 5 × FAD mouse model, it was shown that Aβ can lead to the reduced expression of the kinesin heavy chain, KIF5A, promoting defects in Aβ-induced anterograde transport of mitochondria, a process that can be ameliorated by restoring intracellular KIF5A levels (Wang et al., 2019). Alternatively, Aβ may also interact with the dynein intermediate chain (DIC), disrupting dynein coupling to its adaptor protein, snapin. This process affects the ability of dynein to promote retrograde transport, resulting in impaired retrograde mitochondrial transport in hAPP transgenic mice (Tammineni et al., 2017). These studies suggest that Aβ can affect axonal transport by altering motor proteins levels and functioning.

**Figure 2:**
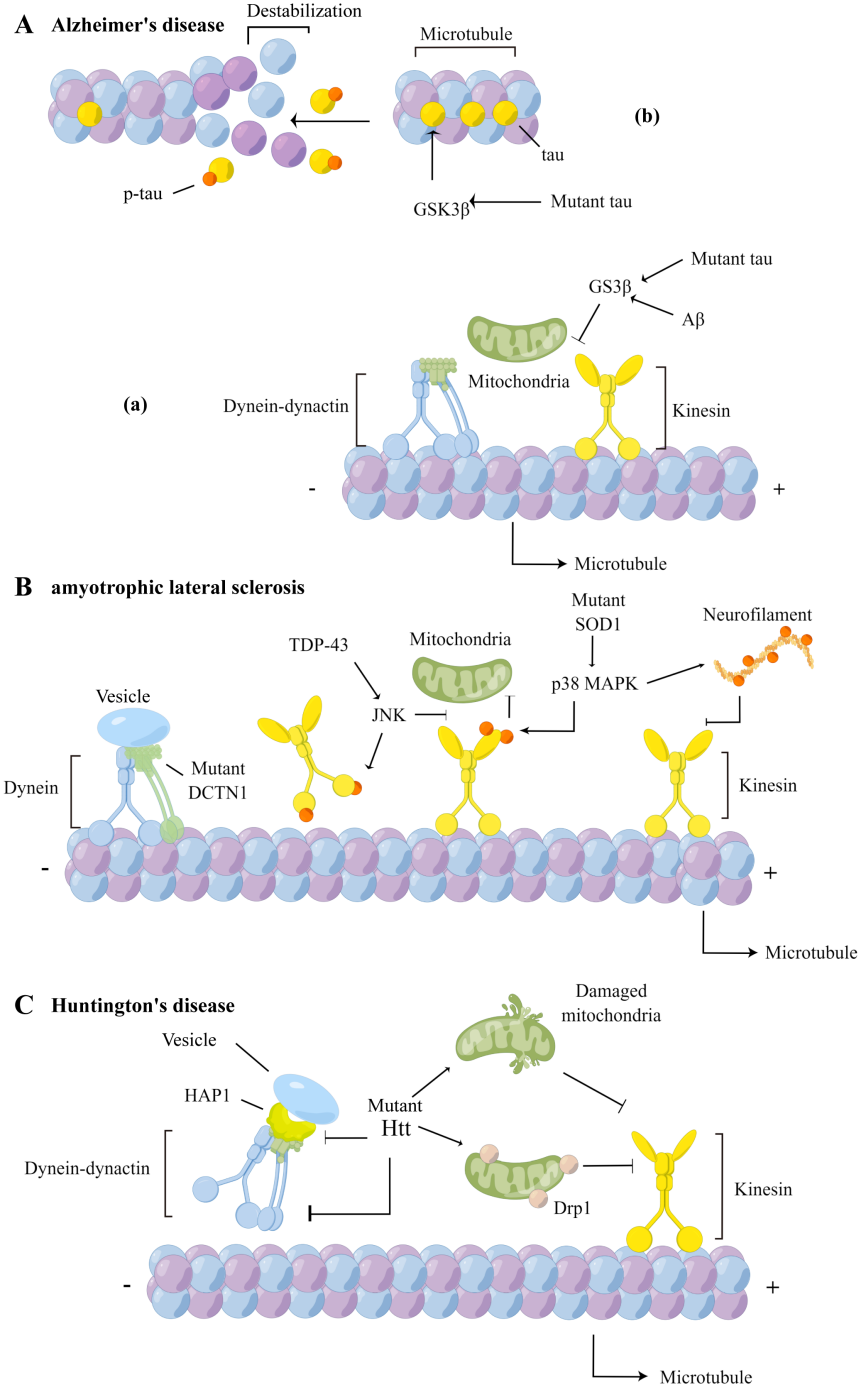
Axonal transport defects in AD, ALS and HD. **(A)** In AD, over-activation of GSK3β increases phosphorylation of tau (b), reducing affinity of tau to microtubules and causing structural changes in microtubules, and affects the binding of cargoes to motor proteins (a). **(B)** In ALS, the DCTN1 mutation affects the retrograde transport of cargoes; TDP-43 inhibits the binding of kinesin to microtubules by affecting JNK activity and phosphorylating the kinesin motor domains; mutant SOD1 activates p38 MAPK, leading to hyperphosphorylation of the kinesin light chain and inhibition of binding to cargo proteins; activated p38 MAPK also phosphorylate NF and inhibit the binding of NF to kinesin; **(C)** In HD, mutant Htt can not only interacts with dynein and HAP1, affecting the retrograde transport function of dynein, but also interact with mitochondria, leading to mitochondrial structural damage and axonal transport impairment. The HAP1-mediated axonal transport is disrupted by mutant Htt as well.

Aβ also affects the post-translational modification of microtubule proteins, altering the dynamic balance of microtubule aggregation and disaggregation, and subsequently causing mitochondrial axonal transport disorders in hippocampal neurons (Kim et al., 2012; Qu et al., 2017), a finding confirmed in 5 × FAD model mice and AD patients (Choi et al., 2017). Not only that, Aβ also affects the normal physiological functioning of the motor adaptor protein of mitochondrial transport, Miro1, by increasing intracellular Ca^2+^ concentrations (Figure 2A-a), a process that further inhibits the bidirectional movement of mitochondria (Guo et al., 2013).

Increased levels of tau phosphorylation is one of the main causes of mitochondrial transport impairment. In normal physiological processes, tau maintains balanced axonal transport by differentially regulating kinesin and dynein (Dixit et al., 2008), and binding and stabilizing microtubules (Tapia-Rojas et al., 2019). In the AD brain, abnormal phosphorylation of tau, especially the hyperphosphorylation of sites near the microtubule-binding sequence, leads to the detachment of tau from microtubules (Figure 2A-b), which causes structural and functional disorders of axons (Martin et al., 2013). It has been found that kinesin-based mitochondrial anterograde transport is inhibited in PC12 cells and mouse cortical neurons after abnormal phosphorylation of the serine 202 and threonine 205 (AT8) of tau (Shahpasand et al., 2012). This implies that hyperphosphorylation of tau can facilitate microtubule structural changes, affecting the role of kinesin in anterograde transport. It has been shown that feedback regulation between abnormally phosphorylated tau (p-tau) and mitochondrial transport processes occurs. On the one hand, p-tau can trap JIP1, a regulator of the kinesin motor complex (Sun et al., 2017), in the cell body, inhibiting the selective binding of JIP1 to Miro1, thus hindering mitochondrial transport in K369I transgenic mice (Ittner et al., 2009). Due to the abnormal distribution of mitochondria within axons, the dynamic balance between tau and microtubule proteins cannot be maintained, further dysregulating tau phosphorylation (Iijima-Ando et al., 2012).

In addition, the effects of Aβ on mitochondrial transport are related to tau. Pathological tau not only impairs mitochondrial axonal transport but also leads to APP transport impairment in cultured hippocampal neurons, increasing APP accumulation *in situ* and inducing downstream effect (Mandelkow et al., 2003). In contrast, when *in vivo* levels of tau are low, so are levels of GSK3β activation (Figure 2A-a). This diminishes Aβ-mediated inhibitory effect on mitochondrial anterograde transport in primary neurons from hAPP mice (Vossel et al., 2015). These findings suggest that pathological tau plays a dominant role in mitochondrial transport impairment in AD.

Taken together, prior studies suggest that tau and Aβ impair the axonal transport of mitochondria. It is unclear whether mitochondria are specifically targeted or pathogenic proteins affects the transport of other organelles as well. Whether Aβ preferentially affects mitochondrial anterograde or retrograde transport is also controversial.

### 2.2. Frontal temporal dementia

FTD is a progressive brain disease characterized by cognitive and behavioral defects that is commonly associated with parkinsonism. Histopathologically, FTD is also a neurodegenerative disease associated with tau pathology, which in familial cases is caused by mutations in gene encoding tau (Zetterberg et al., 2019).

Early studies revealed that both wild-type and genetically mutated tau can affect the axonal transport of multiple cellular components. The pathological mechanism of axonal transport deficits in FTD may be similar to that of AD. In such a mechanism, pathological tau interferes with the binding of kinesin to microtubules or p-tau destabilizes microtubules, affecting anterograde axonal transport. In contrast, in tau P301L knock-in mice in which the mutated protein is transcribed and expressed at physiological levels, there are reduced levels of tau phosphorylation and mitochondrial anterograde axonal transport defects are less severe compared to those of transgenic mouse models overexpressing the human P301L mutation (Gilley et al., 2012). A recent study revealed a R5L mutation in the tau protein inhibits axonal transport by activating protein phosphatase 1 (PP1) (Combs et al., 2021). In hippocampal neurons of rats, R5L tau selectively interacts with PP1α and PP1γ, significantly increasing PP1 activity. Correspondingly, bidirectional axonal transport impairment of synaptophysin caused by R5L tau was ameliorated after knocking down PP1γ expression. In addition, the P301L tau protein has been shown to produce similar effects. These studies suggest that mutant tau may impair axonal transport in neurons by specifically activating PP1γ-dependent pathways.

In summary, in tau pathology-related neurodegenerative diseases, alterations in the structure and function of tau not only directly affect the functioning of axonal transport-related proteins but may also indirectly affect axonal transport in neurons by altering the activities of multiple enzymes.

### 2.3. Amyotrophic lateral sclerosis

Axonal transport deficits are major pathological features of several motor neuron diseases, the most common of which is ALS. ALS is a progressive neurodegenerative disease caused by selective damage to the upper motor neurons in the motor cortex and lower motor neurons in the brainstem and spinal cord. Clinically, patients often present with progressive weakness and atrophy of extremities, chest and bulbar muscle (Štětkářová and Ehler, 2021). The disease is progressive and fatal, usually due to respiratory failure.

Researchers have captured direct evidence of defective retrograde axonal transport of signaling endosomes in ALS using superoxide dismutase 1 (SOD1) transgenic mice (Bilsland et al., 2010; Tosolini et al., 2022). Live cell imaging revealed a reduced number of mitochondria and uneven distribution of fluorescent-labeled mitochondrial anterograde transport in SOD1G93A-expressing cortical neurons versus embryonic motor neurons (De Vos et al., 2007), which further confirmed to be mitochondrial transport deficits in SOD1 mice (Bilsland et al., 2010). These results were confirmed in a SOD1G93A transgenic animal model (Magrané et al., 2012, 2014). Mitochondrial transport defects are not limited to SOD1-associated ALS transgenic models. Overexpression of the mutant vesicle-associated membrane protein B (VAPB), VAPBP56S, selectively impedes mitochondrial anterograde transport activity (Mórotz et al., 2012). Similarly, primary motor neurons overexpressing TDP-43 showed abnormal mitochondrial aggregation (Wang et al., 2013). The above results were verified *in vivo* in the corresponding mouse model (Xu et al., 2010; Sleigh et al., 2020).

The specific mechanism by which axonal transport defects are involved in the pathogenesis of ALS is unclear and may involve several processes, including the mutation of genes encoding axonal transport-related proteins (Puls et al., 2003), altered microtubule stability (Godena et al., 2014), the hyperphosphorylation of motor proteins (Morfini et al., 2013), and weakening of associations between cargoes and motor proteins (Ackerley et al., 2003).

Mutations in genes encoding axonal transport-related proteins are also known to cause ALS. It has been shown that loss-of-function mutations in dynactin 1 (DCTN1) can lead to the development of ALS pathology, mislocalization and aggregation of TDP-43, and obvious upper motor neuron symptoms with a typical progression (Deshimaru et al., 2021; He et al., 2022). Under normal physiological conditions, autophagic vesicles are formed at axon terminals and synapses, and undergo retrograde transport to the cytosol soma mediated by dynein with dynactin along microtubules, for maturation and degradation of abnormal proteins (Evans and Holzbaur, 2019). Mutations in DCTN1 may promote pathological protein accumulation *in situ* and lead to neuronal degenerative processes (Figure 2B). DCTN1 protein mutants, including T1249I, M571T, R785W, and R1101K, have been reported as possible risk factors for ALS (Münch et al., 2004; Mäki-Marttunen et al., 2020); however, further studies are needed to determine their specific roles in the disease. Genome-wide analysis showed that kinesin 5A (KIF5A) mutations are new risk factors for ALS (Nicolas et al., 2018). The conditional knockout of gene encoding KIF5A reduces anterograde axonal transport of NFs (Neurofilaments) within mouse motor neurons, and manifests as neuronal degeneration and paralysis (Xia et al., 2003). Patients with KIF5A gene mutations have an earlier age of disease onset than those without mutations. Baron et al. found that mutations in the KIF5A gene changed the cargo-binding domain of the protein, enhanced protein-RNA interactions, and promoted abnormal anterograde mitochondrial transport in primary mouse cortical neurons, possibly leading to mitochondria accumulate distally (Baron et al., 2022).

TUBA4A, a known ALS gene (Smith et al., 2014), encodes α-tubulin and mutations in T145 and W407 near the α-tubulin binding sequence may affect microtubule heterodimer formation (Liu and Henty-Ridilla, 2022). Similarly, an exome sequencing study confirmed that overexpression of α-tubulin with W407X mutation severely affect inter-microtubule aggregation and track formation (Smith et al., 2014). These results suggest that a normal structure of microtubule is important for neuronal axonal transport (Van Steenbergen et al., 2022). Further, mutations in TUBA4A might result in defective microtubule tracks and possibly contribute to the pathogenesis of ALS.

Microtubule stability is regulated by microtubule-associated proteins (MAPs) such as tau and MAP1B, the latter of which is associated with retrograde mitochondrial transport (Jiménez-Mateos et al., 2006). In clinicopathological biopsy studies, MAP1B was found to be abnormally localized in lumbar spinal cord motor neurons of patients with ALS (Coyne et al., 2014), possibly affecting the degradation and redistribution of damaged mitochondria. Furthermore, researchers found that in neuromuscular junctions of *Drosophila*, transcription and translation of the MAP1B homologue, Futsch, was reduced in the context of TDP-43-induced proteinopathy, and associated with mitochondrial transport impairment, leading to progressive neurodegenerative changes (Bettencourt da Cruz et al., 2005; Coyne et al., 2014). Other findings suggest that the restoration of MAP1B expression has a protective effect on neurons in a *Drosophila* model of ALS (Coyne et al., 2014; Godena et al., 2014). In addition, acetylation modification of α-tubulin proteins is also associated with microtubule stability. Acetylated α-tubulin has been shown to promote kinesin-mediated anterograde transport processes in hippocampal neurons (Hammond et al., 2010). In contrast, TDP-43 can indirectly regulate acetylation of microtubules by altering levels of histone deacetylase 6 (HDAC6) expression, whereas mutant TDP-43 (G287S, N390S and A382T) promoted the deacetylation modification of α-tubulin by HDAC6, which in turn hinders mitochondrial axonal transport (Hubbert et al., 2002; Kim et al., 2010; Fazal et al., 2021).

Phosphorylation of axonal transport-related proteins, including motor proteins and cargoes, regulate the axonal transport functions of cells. p38 mitogen-activated protein kinase (p38 MAPK) and c-Jun N-terminal kinase (JNK) have been shown to be involved in the phosphorylation of related proteins, which in turn regulate axonal transport (Gibbs et al., 2015, 2018). p38 MAPK was shown to be hyperactivated in SOD1 transgenic mice and ALS patients. Activated p38 phosphorylates kinesin at serine 175 and 176, allowing for the inhibition of kinesin-mediated anterograde axonal transport in cultured hippocampal neurons (Figure 2B) (Morfini et al., 2013). p38 MAPK can phosphorylate NF as well, which may affect the binding of NF to motor proteins (Figure 2B), significantly slowing the rate of anterograde axonal transport in cortical neurons (Ackerley et al., 2003). Moreover, hyperactivated p38 MAPK can also lead to impaired retrograde axonal transport of signaling endosomes in primary SOD1G93A motor neurons; whereas acute treatment with p38 MAPKα inhibitors restored the physiological rate of retrograde axonal transport in early symptomatic SOD1G93A mice (Gibbs et al., 2018). These studies suggest that hyperactivated p38 MAPK might selectively hinder bidirectional axonal transport via phosphorylating related motors and cargoes in ALS. In addition, TDP-43 enhances JNK activation (Suzuki and Matsuoka, 2013). It has been shown that activated JNK regulates interaction between kinesin, microtubules and cargoes (Morfini et al., 2006). On the one hand, JNK mediates the phosphorylation of kinesin heavy chain, affecting kinesin’s movement along microtubules (Figure 2B). On the other hand, interactions between kinesin and the cargo adaptor protein JIP1 are disrupted, diminishing the ability of DVGLUT (a synaptic vesicle glutamate transporter) to bind to kinesin via the adaptor protein and carrying out its anterograde movement in *Drosophila* S2 cells (Horiuchi et al., 2007). In addition, JNK affects the binding of JIP3 to DCTN1 and dynein, interfering with the formation of dynein’s retrograde transport complexes. This prevents the binding of dynein to lysosomes and diminishes transport performance, resulting in the terminal accumulations of lysosomes in TgBAC transgenic zebrafish (Cavalli et al., 2005; Huang et al., 2011; Drerup and Nechiporuk, 2013).

In addition, hexanucleotide repeat expansion in the C9orf72 gene is another common genetic cause of ALS. Recent *in vitro* studies have shown that arginine-rich dipeptide repeats could directly block the translocation of dynein and kinesin on microtubule, which hindered mitochondrial transport in the patient stem cell-derived motor neurons (Fumagalli et al., 2021). Above study revealed another potential mechanism underlying the abnormal motor-microtubule bindings, in addition to phosphorylation of motor proteins.

However, Marinkovic et al. considered that the impairments in mitochondrial axonal transport contribute minor to axonal degeneration in ALS models (Marinkovic et al., 2012). Moreover, increasing mitochondrial transport failed to produce a significant therapeutic effect in the dorsal root ganglion neurons isolated from SOD1G93A mice (Zhu and Sheng, 2011). Although mitochondrial transport deficits are involved in the neurodegenerative process, it seems that they are not the primary cause of neuropathology and therefore improving axonal transport deficits may not be an appropriate therapeutic target for ALS.

In summary, there are multiple ways in which axonal transport is affected in the ALS nervous system. Main pathological mechanisms involve downregulation of the expression of motor proteins, phosphorylation of key transport proteins, reduced binding to transport cargoes and changes in microtubule stability. Currently, no conclusive evidence has shown which pathological mechanisms have the greatest impacts on the pathology of ALS; therefore, further studies are needed.

### 2.4. Parkinson’s disease

Lewy body (LB) formation and nigrostriatal dopamine neuron degeneration are the main pathological features of PD. Abnormal aggregation of α-Syn is a major component of LBs (Goedert et al., 2013), and its propagation throughout the PD brain might be supported by axonal transport (George et al., 2013). Studies *in vitro* have shown that α-Syn aggregates could be retrograde transported to the cell body and then deposit there (Pan-Montojo et al., 2012). Human α-Syn pathology progressively spread from the medulla to upper brain regions in a rat model which overexpressed human α-Syn after vagus neuronal injection (Ubeda-Bañon et al., 2014). Whereas partial resection of the vagus resulted in reduced α-Syn aggregates in colon tissues in a novel rat model after intrastriatal injection of preformed fibrils (Wang et al., 2022). Axonal transport may be crucial to disease progression, and the propagation of α-Syn pathology will be halted if the axon is damaged.

However, some investigators have proposed that axonal transport deficits are already present in the early stages of the disease, and has the potential to contribute to neuronal failure in PD (Millecamps and Julien, 2013). Clinical studies have shown that there are decreased levels of kinesin protein in the early stage of PD before alterations in dopaminergic phenotypic markers (such as Tyrosine hydroxylase, TH), a phenomenon that is more pronounced in neurons with α-Syn accumulation (Chu et al., 2012). This phenomenon was reproduced in an A30P rat model of Parkinson’s disease. It implies an interaction between α-Syn pathology and diminishing levels of kinesins in PD. In addition, as kinesin motors supply mitochondria to the axonal compartments and synapses, the reduction in kinesin levels further led to impaired anterograde axonal transport of mitochondria and ATP deficit in human iPSC (induced pluripotent stem cell)-derived PD neurons carrying α-Syn gene duplication (Prots et al., 2018; Sabui et al., 2022). The understanding of pathological mechanisms of reducing kinesin expression in facilitating restoration of anterograde transport will improve spatial abnormal distribution and dysfunction of mitochondria in PD. A clinical study showed that activated 5′-monophosphate-activated protein kinase (AMPK) inhibits expression of kinesin at the transcriptional level (Oliveras-Ferraros et al., 2009). Furthermore, pathological α-Syn can depress the latter’s inhibitory effect on AMPK by inhibiting PI 3-kinase enhancer (PIKE) activity, leading to AMPK overactivation and eventually neuronal death (Kang et al., 2017). Thus, the existing pathological α-Syn may greatly affect the efficiency of anterograde axonal transport through downregulating kinesin expression via indirectly activating AMPK since early stage of PD.

Unlike kinesin, studies showed that the levels of dynein were upregulated in the striatum of rat models at early time points (Chung et al., 2009; Yang et al., 2022), and its reductions were only observed at late PD stages, which was significantly greater in nigral neurons containing α-Syn inclusions (Chu et al., 2012). Co-expressions of LBs with DCTN1 and DYNLT3 (dynein light chain Tctex type 3, a subunit contributing to dynein cargo binding specificity) were identified in the substantia nigral neurons of PD patients (Lo et al., 2007; Chu et al., 2012; Shen et al., 2018). Moreover, α-Syn aggregates reduced the retrograde transport of LC3+ autophagosomes, Rab7+ and TrkB+ endosomes in rat primary midbrain neurons (Volpicelli-Daley et al., 2014; Koch et al., 2015), which is jointly mediated by a transport complex formed by dynein, DCTN1 and snapin under physiological conditions. Though upregulation of dynein expression perhaps reflect its enhanced function at early stage of PD (Steinberg, 2011), promoting protein aggregates clearance (Ripon et al., 2020), α-Syn aggregates may interfere with partial retrograde processes via affecting the formation of dynein transport complexes, disturbing extracellular signaling and activity of mitophagy (Volpicelli-Daley et al., 2014; Koch et al., 2015; Boecker et al., 2021; Wang et al., 2022). Recent studies did not reveal a direct effect of α-Syn aggregation on the reduced expression of dynein, and the changes on levels of dynein possibly due to diffused neurodegeneration at late stage of PD.

Furthermore, the axonal microtubule-associated protein tau, known to selectively hinder kinesin-based mitochondrial transport in mouse neurons (Dixit et al., 2008), was significantly increased in human iPSC-derived neurons introduced with α-Syn mutants E46K and E57K, with enriched levels of phosphorylation (Prots et al., 2018). Reportedly, phosphorylation regulates the function of tau (Johnson and Stoothoff, 2004), thus influencing microtubule stability. Since Lewy bodies contain not only α-Syn but also tau (Miller et al., 2022), and an interaction between tau and α-Syn has also been reported (Roy and Jackson, 2014), the α-Syn aggregates could mediate tau phosphorylation via activated protein kinase A and GSK3β (Grassi et al., 2019), resulting in microtubule depolymerization and reduced axonal transport.

To sum up, axonal transport deficits are early pathological events in PD, including reduced kinesin protein expression, hindered formation of dynein transport complexes and microtubule depolymerization. Most of above mechanisms are directly or indirectly mediated by α-Syn pathology, which further influence cell activities without correct localization, such as mitophagy and ATP supplement (Prots et al., 2018; Boecker et al., 2021).

### 2.5. Huntington’s disease

Polyglutamine diseases are a group of neurodegenerative disorders caused by the repetitive amplification of CAG sequences. The diseases include Huntington’s disease (HD), Kennedy’s disease, and some spinal cerebellar ataxias. Repeatedly amplified pathogenic gene transcription results in the translation of pathogenic proteins with polyglutamine chains that aggregate within cells, affecting normal physiological functioning in the cytoplasm, including axonal transport (Lee et al., 2004; Chang et al., 2006).

The molecular mechanisms by which pathogenic proteins disrupt axonal transport in this class of neurodegenerative diseases are unknown; however, the pathogenesis of HD may be mediated via two mechanisms. Firstly, mutant Huntingtin (Htt), a pathogenic protein of Huntington’s disease, may play a role in dynein-mediated retrograde axonal transport (Figure 2C) (Illarioshkin et al., 2018). Mutant Htt can not only directly interact with dynein, but also indirectly affects retrograde transport of brain-derived neurotrophic factor-containing vesicles and Golgi via Huntingtin-associated protein 1 (HAP1) with DCTN1 in mouse mutant STHdh cells (Pardo et al., 2010; Saudou and Humbert, 2016). Contrarily, the complex formed by HAP1 and dynactin is rapidly detached from microtubules in the absence of mutant Htt, a process that results in the restoration of neurotrophic factor’s axonal transport within 109Q cells treated with wildtype-Full length-Htt (Gauthier et al., 2004).

In addition, accumulated mutant Htt interacts directly with mitochondria (Figure 2C), leading to mitochondrial structural damage and anterograde axonal transport impairment (Shirendeb et al., 2012). In both pathological HD tissues and primary neurons of BACHD mice, mutant Htt was found to interact with the mitochondria-associated protein Drp1, enhancing its enzymatic activity, altering mitochondrial dynamics and hindering anterograde axonal transport.

To summarize, the pathological Htt protein in ALS interacts with motors and adaptors, directly and indirectly reduced vesicles and organelles, such as Golgi and mitochondria, axonal movement, further disrupting intracellular localization and normal physiological functioning.

## 3. Conclusions and perspective

Progressive accumulation of specific proteins in different types of neurons and triggering neurodegeneration are common features in several neurodegenerative diseases. Whether abnormal protein accumulation is a cause or a consequence of axonal transport defects, or whether they are mutually causal, is a subject of debate. There is no doubt that impaired axonal transport is an early important pathological event in several neurodegenerative diseases. Mechanisms by which axonal transport is impaired vary among diseases. Nonetheless, the following pathways have been concluded in this review: (1) motor protein changes including expression level and post-translational modification alteration; (2) changes in microtubules including reducing stability and disrupting tracks; (3) changes in cargo including diminishing binding to motor proteins (Table 1). Among these, mutations in genes encoding proteins involved in axonal transport support the idea that defects in intracellular axonal transport accelerate neurodegeneration (Kieran et al., 2005; Millecamps and Julien, 2013; Nicolas et al., 2018). To summarize, the causal relationship between impaired axonal transport and abnormal protein aggregation may vary among diseases. Studying mechanisms by which axonal transport defects contribute to various diseases has the potential to facilitate the development of novel drugs and improve clinical guidelines.

**Table 1:** Mutations in axonal transport deficits of neurodegenerative diseases.

Gene	Function	Mutant	Affected axonal transport process	References
**Alzheimer’s disease**				
APP	Positively influence metabolism after non-amyloidogenic processing	Tg2576	Anterograde axonal transport of mitochondria	Calkins et al. (2011)
		5 × FAD	Kinesin expression	Wang et al. (2019)
			Microtubule stabilization	Choi et al. (2017)
		J20	Motor adaptor protein binding to motor	Tammineni et al. (2017)
MAPT	Helps assembly, stabilization, and modulation of microtubules	2A, 3A, 3D	Microtubule stabilization	Shahpasand et al. (2012)
		K369I	Cargo binding to motor proteins	Ittner et al. (2009)
**Frontal temporal dementia**				
MAPT	Helps assembly, stabilization, and modulation of microtubules	P301L	Anterograde axonal transport of mitochondria	Gilley et al. (2012)
		R5L	Axonal transport of synaptophysin via activation of PP1	Combs et al. (2021)
**Amyotrophic lateral sclerosis**				
SOD1	Protect the cell from reactive oxygen species toxicity	G93A	Retrograde axonal transport of signaling endosomes via activation of p38 MAPK	Gibbs et al. (2018); Tosolini et al. (2022)
			Anterograde axonal transport of mitochondria	Magrané et al. (2014)
			Phosphorylate kinesin via activation of p38 MAPK	Morfini et al. (2013)
VAPB	Tethering molecule at the membrane contact sites between the endoplasmic reticulum and intracellular organelles	P56S	Anterograde axonal transport of mitochondria	Mórotz et al. (2012)
DCTN1	Cofactor of cytoplasmic dynein that mediate most retrograde axonal transport	T1249I M571T R785W R1101K	Unknown effect but shown to affect retrograde axonal transport	Münch et al. (2004); Mäki-Marttunen et al. (2020)
KIF5A	Heavy chain of kinesin that mediate diverse anterograde axonal transport	KIF5A^ΔExon27^	Cargo binding to motor proteins	Baron et al. (2022)
TUBA4A	Microtubule component that maintain neuronal process and synaptic function	W407X	Microtubule stabilization	Smith et al. (2014)
TDP-43	Repressor of cryptic exon inclusion during RNA splicing	G287S N390S A382T	Deacetylation modification of α-tubulin via HDAC6	Hubbert et al. (2002); Kim et al. (2010); Fazal et al. (2021)
C9orf72	A predicted guanine exchange factor	Hexanucleotide repeat expansion	Translocation of dynein and kinesin on microtubule	Fumagalli et al. (2021)
**Parkinson’s disease**				
SNCA	Synaptic transmission	A30P	Decrease kinesin expression	Chu et al. (2012)
		E46K	Anterograde axonal transport of mitochondria	Prots et al. (2018)
		E57K	Phosphorylation of tau via GSK3β and stabilization of microtubules	Prots et al. (2018); Grassi et al. (2019)
**Huntington’s disease**				
HTT	Synaptic neurotransmission	polyQ expansion	Retrograde transport of BDNF via HAP1	Pardo et al. (2010)
			Mitochondrial structural damage and anterograde axonal transport via activation of Drp1	Shirendeb et al. (2012)

APP: amyloid precursor protein; MAPT: microtubule associated protein tau; PP1: protein phosphatase 1; SOD1: superoxide dismutase 1; p38 MAPK: p38 mitogen-activated protein kinase; VAPB: vesicle-associated membrane protein B; DCTN1: dynactin subunit 1; KIF5A: kinesin family member 5A; TDP-43: TAR DNA binding protein 43; HDAC6: histone deacetylase 6; SNCA: α-synuclein; GSK3β: glycogen synthase kinase 3β; HTT: huntingtin; BDNF: brain-derived neurotrophic factor; HAP1: huntingtin-associated protein 1; Drp1: dynamin-related protein 1.

## Author contributions

XY wrote the first version of the manuscript. XC conceptualized the review. ZM and PL were in charge of literature searching. YX revised the manuscript. All authors contributed to the article and approved the submitted version.

## Funding

This work was supported by grant from the National Natural Science Foundation of China (NSFC Project, Nos. 81873734 and 81974200).

## Conflict of interest

The authors declare that the review was conducted in the absence of any commercial or financial relationships that could be construed as a potential conflict of interest.

## Publisher’s note

All claims expressed in this article are solely those of the authors and do not necessarily represent those of their affiliated organizations, or those of the publisher, the editors and the reviewers. Any product that may be evaluated in this article, or claim that may be made by its manufacturer, is not guaranteed or endorsed by the publisher.
